# Supplementary material for: 13R,20-Dihydroxydocosahexaenoic Acid, a Novel Dihydroxy- DHA Derivative, Inhibits Breast Cancer Stemness through Regulation of the Stat3/IL-6 Signaling Pathway by Inducing ROS Production
Source: Antioxidants (Basel). 2021 Mar 15;10(3):457. doi: 10.3390/antiox10030457 (PMC7999786; doi:10.3390/antiox10030457)
Supplement: Supplementary file 1 [file antioxidants-10-00457-s001.pdf]

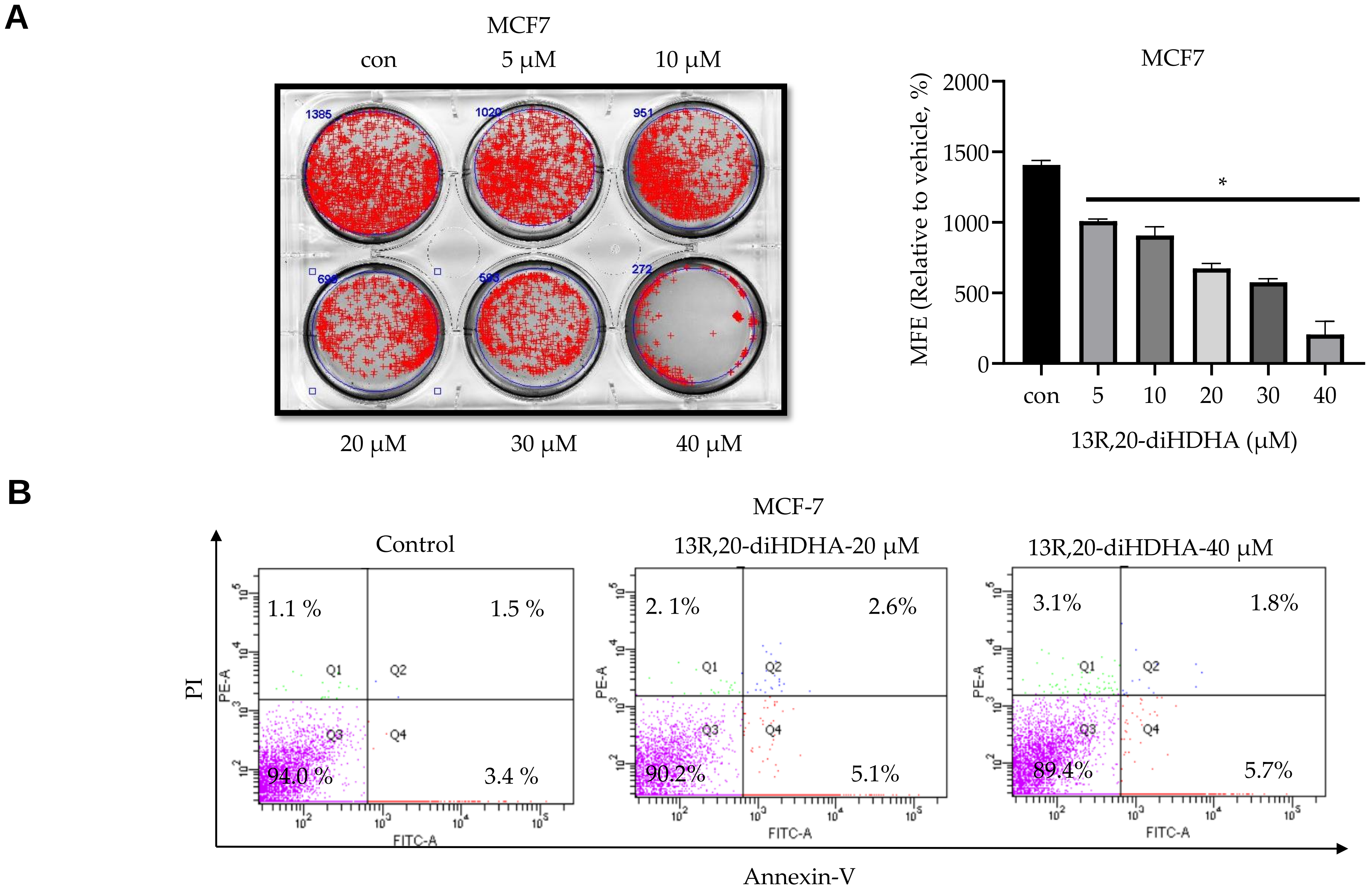

Supplementary Figure S1. The effect of 13R,20-diHDHA on mammospheres formation and multiple cancer hallmarks in breast cancer cell lines. (**A**) The mammospheres formation efficiency (MFE) was decreased by 13R,20-diHDHA treatment. Mammospheres derived from MCF-7 cells were cultured for 7 days in the presence of 13R,20-diHDHA (5, 10, 20, 30, and 40 µM) or DMSO. Image shows the sizes of representative mammospheres, as obtained by microscopy (scale bar: 100 µm). (**B**) 13R,20-diHDHA does not induce significant apoptosis of MCF-7 cells. Apoptosis was etermined using Annexin V/propidium iodide (PI) staining and FACS.

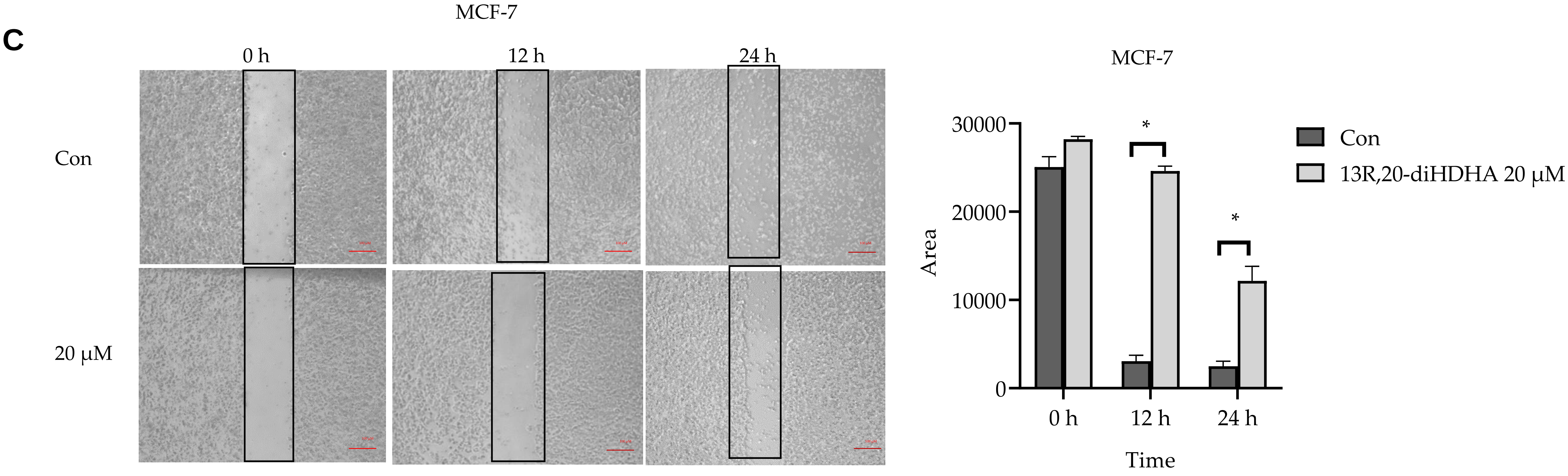

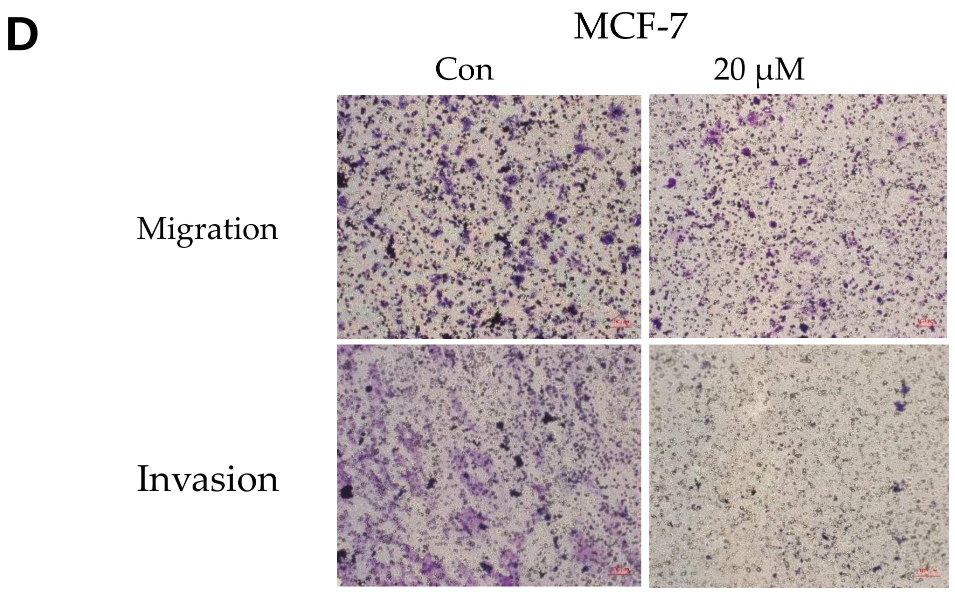

Supplementary Figure S1. (**C**) The migration of MCF-7 cells treated with or without 13R,20-diHDHA (RPMI1640/0.5% FBS) was imaged at 0, 12, and 24 h by a scratch assay (scale bar: 100 μm), and the area was calculated using the Image J software. (**D**) The cell migration (without Matrigel) and invasion (with Matrigel) of MCF-7 cells exposed to 13R,20-diHDHA were determined by transwell assays (scale bar: 100 μm). Representative colony formation data were collected. The data from triplicate experiments are presented as the mean ± SD. * $p < 0.05$ versus the DMSO-treated control group.

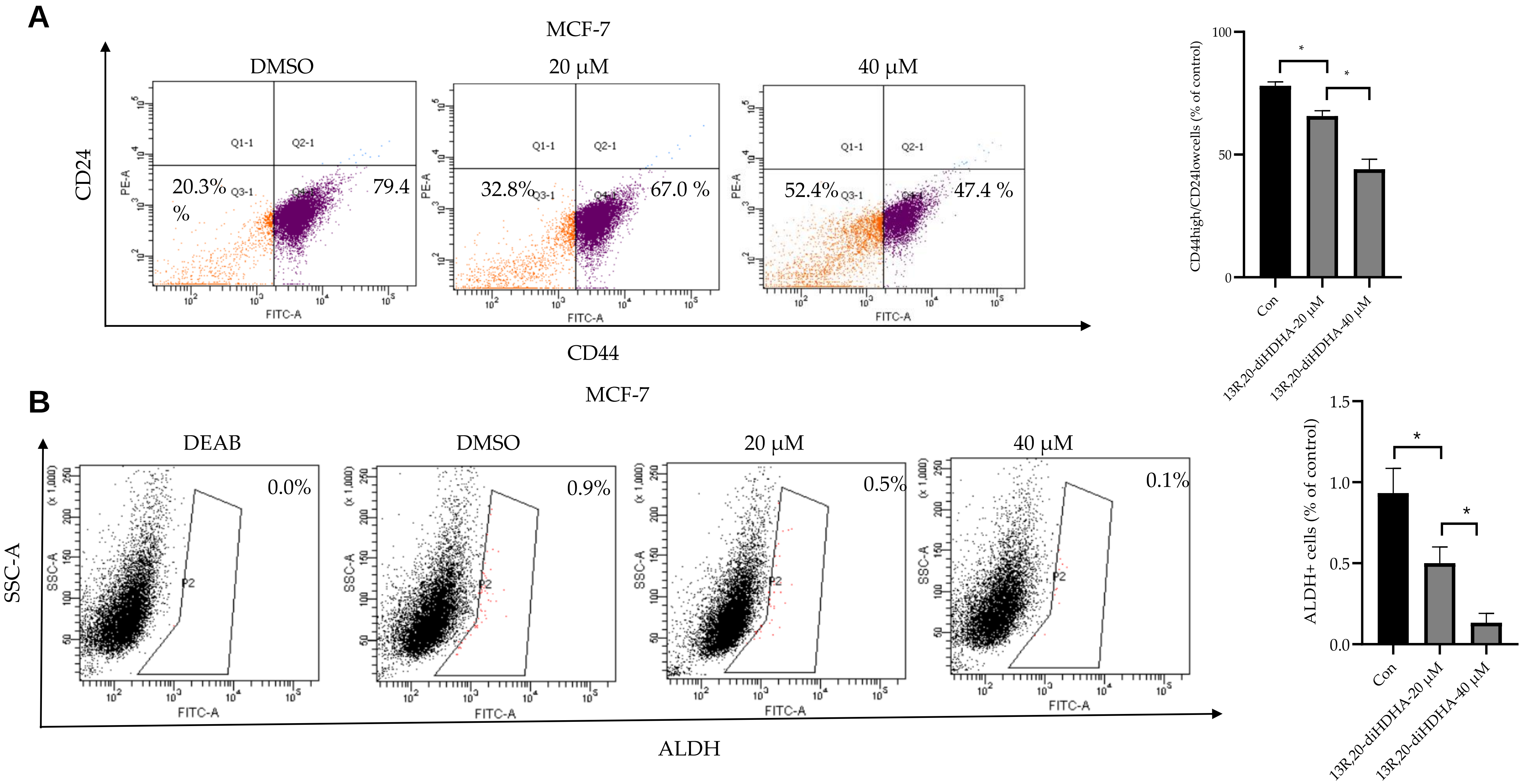

Supplementary Figure S2. The effect of 13R,20-diHDHA on the CD44^{high}/CD24^{low} and aldehyde dehydrogenase (ALDH)-positive cell proportions. (A) The CD44^{high}/CD24^{low} cell populations of MCF-7 cells treated with 13R,20-diHDHA (20 or 40 μM) or DMSO for 24 h were analyzed by FACS. The gating was based on binding of a control antibody. (B) 13R,20-diHDHA decreased the ALDH-positive cell population, as detected with an ALDEFLUOR™ kit (Vancouver, BC, Canada). Breast cancer cells were treated with 13R,20-diHDHA (20 or 40 μM) for 24 h and subjected to FACS analysis. Representative flow cytometric data are shown. The left panel shows the ALDH-positive population in the presence of the ALDH inhibitor, DEAB, and the right panel represents the ALDH-positive population without DEAB. The data from triplicate experiments are presented as the mean ± SD. * $p < 0.05$ versus the DMSO-treated control group.

A

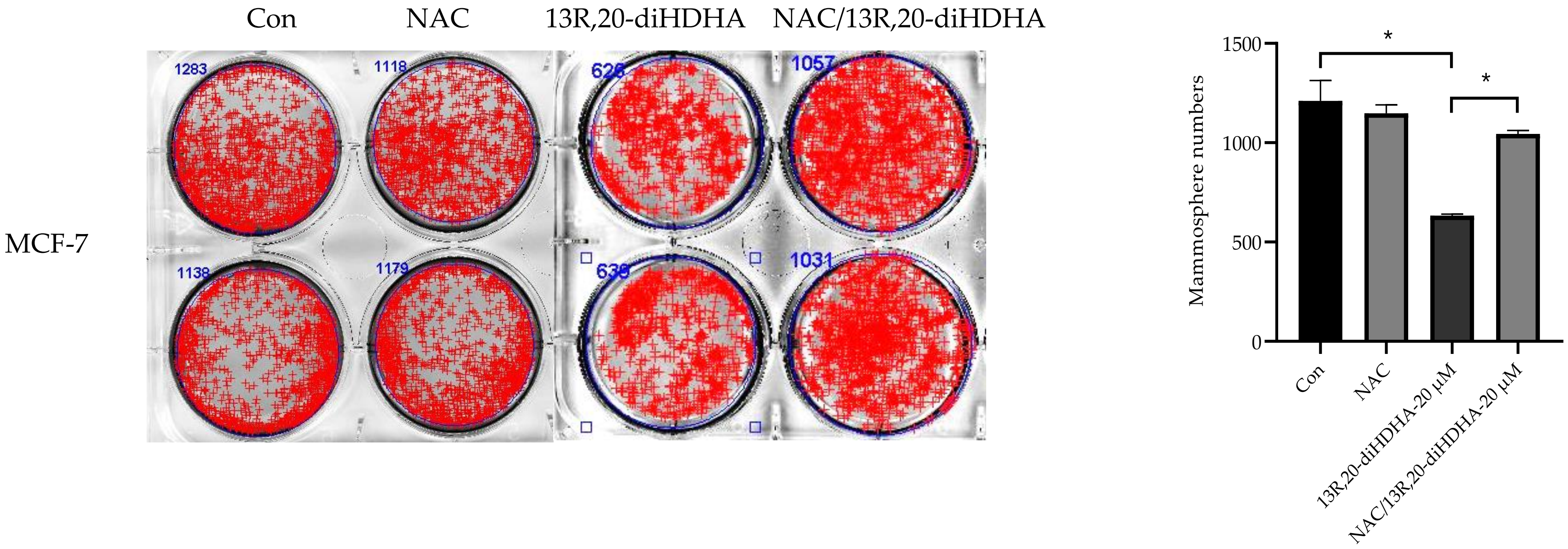

Supplementary Figure S3. Effect of 13R,20-diHDHA-induced ROS generation on mammospheres formation. (**A**) Mammospheres were pretreated with/without NAC (10 mM) for 1 h prior to treatment with 20 μM 13R,20-diHDHA. After 7 days, mammospheres formation was determined. Representative images were obtained under 10x magnification (scale bar: 100 μm).

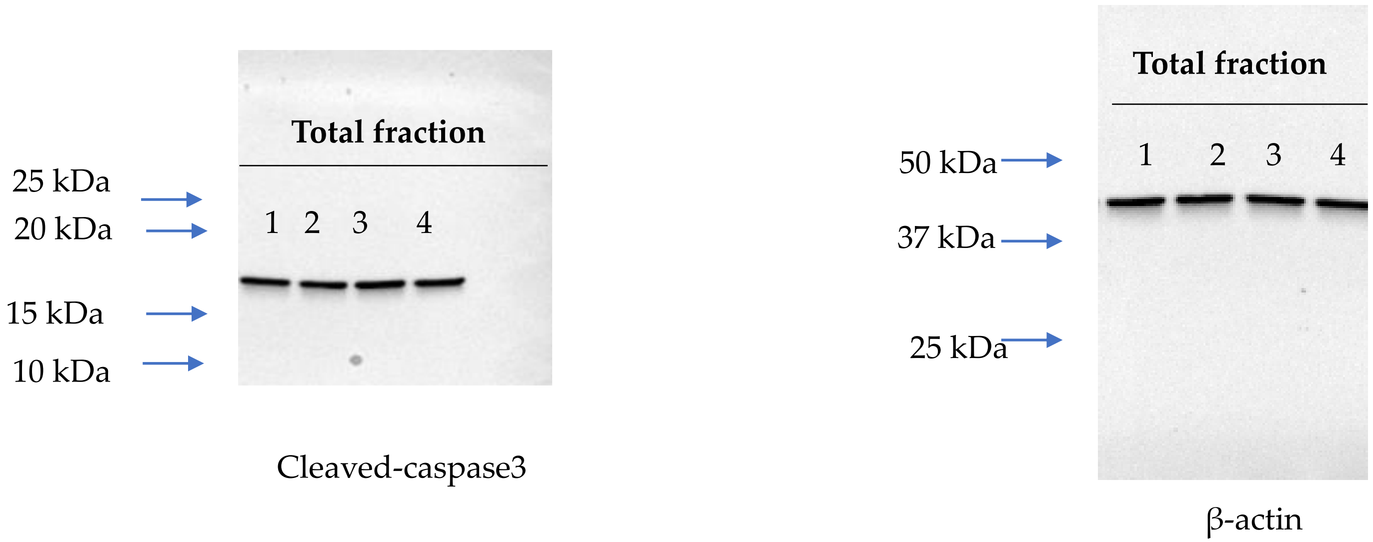

1:MDA-MB-231, control

2:MDA-MB-231, 13R,20-diHDHA

3:MCF-7, control

4:MCF-7, 13R,20-diHDHA

Figure 2E

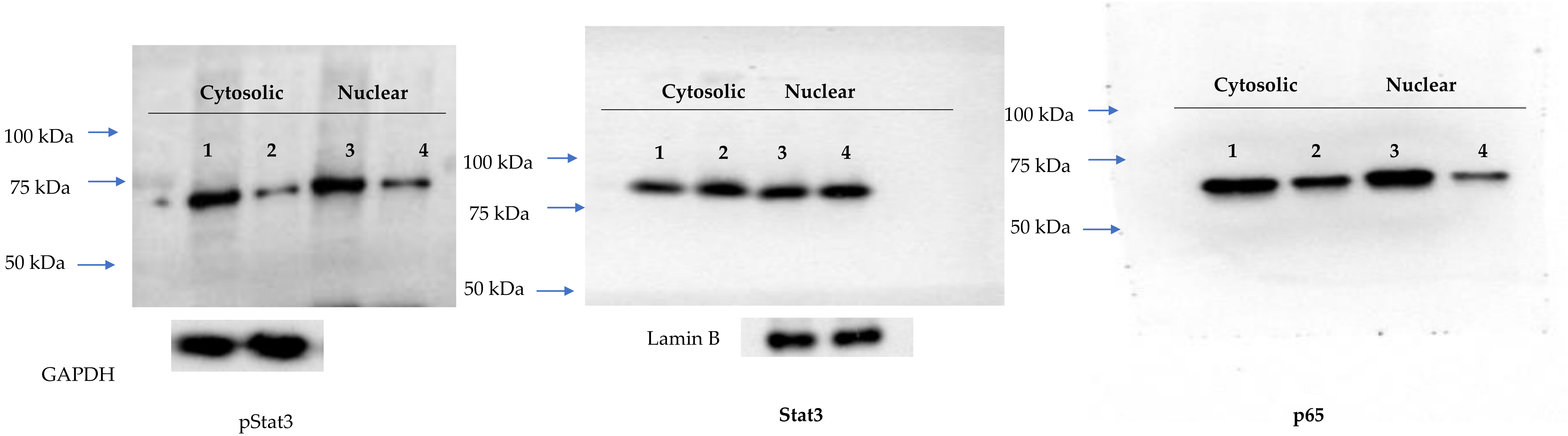

1:MDA-MB-231, control; Cytosolic

2:MDA-MB-231, 13R,20-diHDHA; Cytosolic

3:MDA-MB-231, control; Nuclear

4:MDA-MB-231, 13R,20-diHDHA; Nuclear

Figure 5A

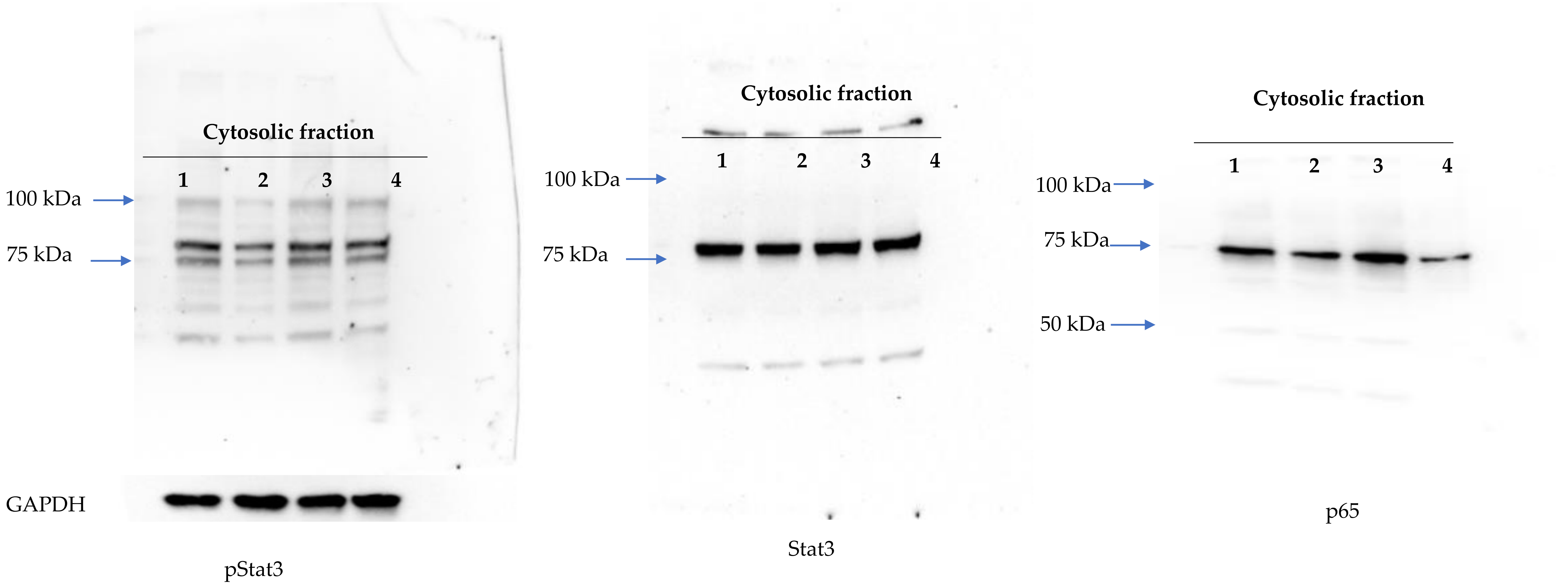

1: MDA-MB-231, control

2: MDA-MB-231, 13R,20-diHDHA

3: MDA-MB-231, NAC

4: MDA-MB-231, NAC/13R,20-diHDHA

Figure 5B

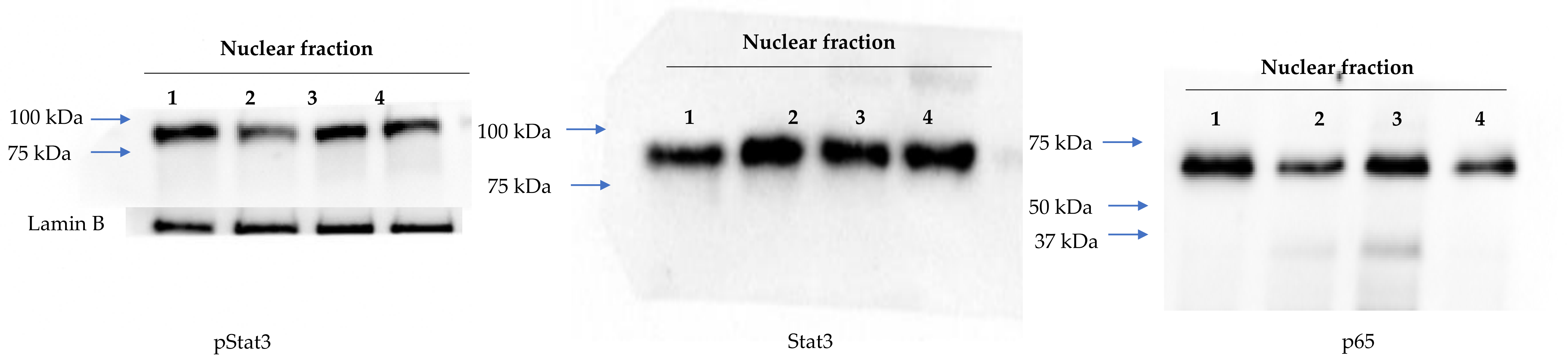

1: MDA-MB-231, control

2: MDA-MB-231, 13R,20-diHDHA

3: MDA-MB-231, NAC

4: MDA-MB-231, NAC/13R,20-diHDHA

Figure 5B

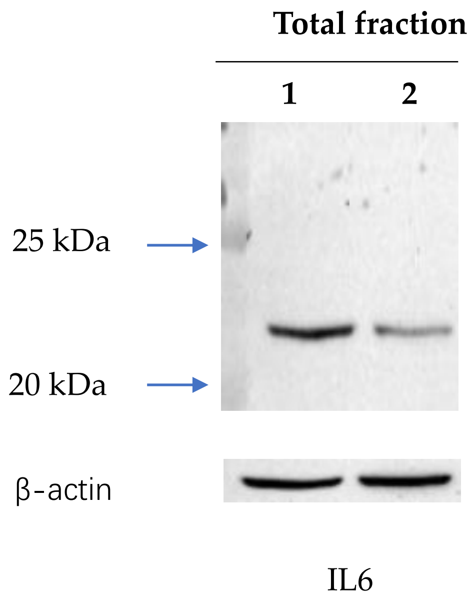

1: MDA-MB-231, control

2: MDA-MB-231, 13R,20-diHDHA

Figure 5D
